# Different patients, different preferences: A multicenter assessment of patients' personality traits and anxiety in shared decision making

**DOI:** 10.1002/cam4.4667

**Published:** 2022-03-24

**Authors:** Anja K. Köther, Björn Büdenbender, Britta Grüne, Sonja Holbach, Johannes Huber, Nicolas von Landenberg, Julia Lenk, Thomas Martini, Maurice S. Michel, Maximilian C. Kriegmair, Georg W. Alpers

**Affiliations:** ^1^ Department of Psychology, School of Social Sciences University of Mannheim Mannheim Germany; ^2^ Department of Urology and Urosurgery, University Medical Center Mannheim, Medical Faculty Mannheim Heidelberg University Mannheim Germany; ^3^ Department of Urology, Caritas St Josef Medical Center University of Regensburg Regensburg Germany; ^4^ Department of Urology Philipps‐University Marburg Marburg Germany; ^5^ Department of Urology Medical Faculty Carl Gustav Carus, TU Dresden Dresden Germany; ^6^ Department of Urology, Marien Hospital Ruhr‐University Bochum Herne Germany; ^7^ Urological Hospital Munich‐Planegg Planegg Germany; ^8^ Department of Urology University Hospital Ulm Ulm Germany

**Keywords:** behavioral science, ethical considerations, psychosocial studies, urological oncology

## Abstract

**Objective:**

Patient‐centered care and shared decision making (SDM) are generally recognized as the gold standard for medical consultations, especially for preference‐sensitive decisions. However, little is known about psychological patient characteristics that influence patient‐reported preferences. We set out to explore the role of personality and anxiety for a preference‐sensitive decision in bladder cancer patients (choice of urinary diversion, UD) and to determine if anxiety predicts patients' participation preferences.

**Methods:**

We recruited a sample of bladder cancer patients (*N* = 180, primarily male, retired) who awaited a medical consultation on radical cystectomy and their choice of UD. We asked patients to fill in a set of self‐report questionnaires before this consultation, including measures of treatment preference, personality (BFI‐10), anxiety (STAI), and participation preference (API and API‐Uro), as well as sociodemographic characteristics.

**Results:**

Most patients (79%) indicated a clear preference for one of the treatment options (44% continent UD, 34% incontinent UD). Patients who reported more conscientiousness were more likely to prefer more complex methods (continent UD). The majority (62%) preferred to delegate decision making to healthcare professionals. A substantial number of patients reported elevated anxiety (32%), and more anxiety was predictive of higher participation preference, specifically for uro‐oncological decisions (*β* = 0.207, *p* < 0.01).

**Conclusions:**

Our findings provide insight into the role of psychological patient characteristics for SDM. Aspects of personality such as conscientiousness influence treatment preferences. Anxiety contributes to patients' motivation to be involved in pertinent decisions. Thus, personality and negative affect should be considered to improve SDM.

## Graphical abstract

We set out to explore the role of personality and anxiety for a preference‐sensitive decision in a multicenter assessment of bladder cancer patients (*N* = 180; choice of urinary diversion, UD). Most patients indicated a clear preference for one of the treatment options (44% continent UD, 34% incontinent UD) and patients who reported more conscientiousness were more likely to prefer more complex treatment methods (continent UD). Moreover, anxiety was predictive of higher participation preference, specifically for uro‐oncological decisions.

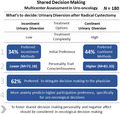

## 1 BACKGROUND

Patient‐centered care and shared decision making (SDM) are nowadays generally recognized as the gold standard for medical consultations. SDM is a collaborative approach to communication in which physicians and patients interact as partners in the decision making process.^1^ In SDM, physicians support patients in achieving an informed treatment preference by incorporating patient preferences and values in the discussion.^2^ The goal of SDM is to identify the treatment option with the best benefit‐harm ratio for the individual patient while providing the best alignment with their preferences. This makes SDM especially relevant for preference‐sensitive decisions, that is, decisions with equivalent treatment options or similar benefit‐harm ratios but different consequences for patients.^3^ Such preference‐sensitive decisions are particularly common in oncology.

Patient preferences are likely to be influenced by psychological characteristics such as anxiety or personality traits.^4^, ^5^ However, for SDM, there is a substantial lack of available information on how patient's psychological characteristics influence patient‐reported preferences (i.e., treatment preference and participation preference).

### 1.1 Anxiety and SDM

Awareness of the emotional needs of cancer patients has steadily increased. Nevertheless, many oncological patients experience emotional distress.^6^ In uro‐oncological samples, about 30% of patients typically report elevated anxiety levels.^7^ While recent studies have established a link between negative affect and adverse decision outcomes,^7^, ^8^ an association between negative affect and participation preference has not been demonstrated.^9^ Moreover, the influence of anxiety on treatment preferences has not been examined thoroughly.

In general, high levels of anxiety may impair decision making capacity^10^, ^11^ and alter risk appraisal.^12^ In recent research, during the COVID‐19 pandemic, we found that anxiety can increase participation preference when individuals are asked about a specific condition relevant to them.^13^ This aligns with literature on anxiety‐related coping styles in oncology.^14^ Anxiety typically activates worries and rumination. In an attempt to re‐establish a feeling of control, patients may express a stronger preference for participation to address disease‐related concerns in the consultation. However, more evidence is needed to fully understand which impact anxiety has on patients' preference for active participation in decision making.

### 1.2 Personality and SDM

Several studies have addressed the role of personality traits in the clinical setting,^15^, ^16^ and personality traits have previously been linked to uro‐oncological treatment and decision making outcomes.^7^, ^17^ The most fruitful approach to describe facets of personality is the five‐factor model,^18^ which defines five global domains of personality: openness to experience, conscientiousness, extraversion, agreeableness, and neuroticism. Individuals with high openness are typically autonomous, physically active, and willing to take risks. Conscientiousness is linked to self‐discipline and focus.^18^ We expected personality traits to impact patients' treatment preferences. As personality reflects communicative tendencies, we also expected it to be relevant for the decision‐making interaction.^5^ However, due to a lack of literature on the association between personality and participation preference, we refrained from establishing directed hypotheses.

### 1.3 Decision context

Research indicates that cancer patients' participation preference is not very stable across time^19^ and may vary, depending on the context patients are asked about.^13^ Thus, it is crucial to re‐evaluate patients' participation preferences for specific decision contexts.

### 1.4 Urinary diversion after radical cystectomy

A prime example of a preference‐sensitive decision context is the decision on the type of urinary diversion (UD) after radical cystectomy, the standard treatment in patients with muscle‐invasive bladder cancer.^20^, ^21^ During radical cystectomy, the patient's bladder is removed, and a new urinary diversion (UD) needs to be created. There are several methods of UD in two main categories: continent vs. incontinent UD.^22^ Both offer satisfiable quality of life and long‐term functionality, and most patients who undergo radical cystectomy are eligible for either one.^20^, ^21^

Each method has significant consequences for the patient and comes with specific advantages and disadvantages.^20^, ^21^, ^22^ The construction of an incontinent UD is less invasive and generally associated with a shorter operative time.^20^, ^22^ However, due to the necessity of an external stoma and urine collection device, it comes with severe changes in body image, which may impair patients.^20^, ^22^ On the other hand, continent UD requires a lengthier operation, entails a greater risk for nocturnal urinary incontinence, and involves an extensive rehabilitation process that patients need to follow conscientiously. Yet, this intervention provides patients to be independent of external stoma devices.^20^, ^22^

While there is comprehensive literature and consensus on relevant medical factors that influence how suitable each method is for a patient,^22^ a good fit likely depends on psychological characteristics.^21^, ^22^ However, the relationship between psychological patient characteristics (i.e., five‐factor personality traits and anxiety) and patient‐reported preferences has not been researched extensively. Consequently, this makes bladder‐cancer patients' choice for UD the ideal context to address these research gaps.

We aimed to explore the influence of psychological patient characteristics on patient‐reported preferences in two ways: First, we aimed to explore differences in participation preference, anxiety, and personality traits for individuals who preferred continent vs. incontinent UD. Specifically, we expected lower openness to be associated with less complex treatment methods (incontinent UD) and less conscientious individuals to prefer treatments that require a less intense and rigorous personal contribution for rehabilitation (as in incontinent UD). Second, we aimed to explore correlates for uro‐oncological decision making. Moreover, we expected anxiety to predict specific participation preference but had no hypothesis on the effect's direction.

## 2 METHODS

### 2.1 Participants

We conducted a multicenter study at urology departments in six German hospitals. We recruited patients admitted for surgical consultation before radical cystectomy (September 2019 through July 2021). During the pre‐treatment consultation, physicians, and patients typically decide on the most suitable type of UD. Data collection took place before a consultation preceding treatment. Participation was not reimbursed.

Patients were eligible to participate when they gave written informed consent and met the following inclusion criteria: age ≥ 18, proficiency in German. We obtained ethical approval for the study from the research ethics committee of the Medical Faculty of Mannheim of the University of Heidelberg (MA‐2019‐727N). We recruited a large convenience sample of *N* = 191 patients. The majority (94.76%, *N* = 181) completed all measures. Ten participants (5.24%) were considered non‐completers and excluded from analyses due to substantial missing data (≥50%). One patient was excluded due to dementia diagnosis. The final sample was primarily male (75.6%) and elderly (*M* = 68.77, SD = 9.28; range 46 to 86).

### 2.2 Data collection and measures

Nurses identified eligible patients after their admission to the clinic, before the pre‐treatment consultation. After giving informed consent, they were invited to fill in a survey while waiting for their consultation. The survey consisted of the following self‐report questionnaires in the given order: sociodemographic characteristics, a short measure of personality (Big Five Inventory; BFI‐10),^23^ the State–Trait Anxiety Inventory (STAI),^24^ patient‐reported preference for UD, and patients' desire for autonomy in decision making (Autonomy Preference Index, API,^25^ followed by uro‐oncological case vignettes, API‐Uro^26^). In addition, we obtained further clinical information from the patients' electronic health records.

#### 2.2.1 Sociodemographic and medical variables

We collected sociodemographic characteristics (age, gender, education, occupational status, nationality, marital status, offspring). In addition, we collected information on patients' tumor stage from their medical records.

#### 2.2.2 Patient‐reported preference for UD

Patients were given a questionnaire asking them which method of UD they preferred. In the questionnaire, patients were informed that there are several different methods of UD which entail different possible advantages and disadvantages. They were then asked for an initial assessment of which of the four most common methods of UD they favored at that time (before their medical consultation): that is, orthotopic neobladder (continent), continent catheterizable pouch (continent), ileal‐conduit (incontinent), or ureter skin fistula (incontinent). To ensure that patients fully understood the essence of each method, each one was illustrated with a picture and explained in a brief text. It was also clarified that patients' answers in the questionnaire represented only a first preference and were not a decision on their pending operation.

#### 2.2.3 Participation preference

To assess participation preference, we first asked patients to fill in the validated German version of the Autonomy Preference Index (API).^25^, ^27^ The API is a well‐validated and commonly used self‐report instrument. It consists of two subscales: information seeking (seven items; α = 0.87) and generic decision making (API generic; four items; α = 0.81). Items are coded on a 5‐point Likert scale, with response choices ranging from (0) “strongly disagree” to (4) “strongly agree.” Items on the decision making subscale are inverted. Sum scores are calculated for both scales separately, with higher scores representing a stronger participation preference.

The two subscales were followed by the three original API case vignettes on upper respiratory tract illness, high blood pressure, and myocardial infarction.^27^ On these original API case vignettes, patients indicate nine different decisions (three for each vignette) who should make the corresponding decision on a 5‐point Likert scale ranging from (1) “The physician alone” to (5) “The patient alone.” On each item, scores of three indicated a preference for equally shared decisions. A maximum sum score of 45 indicates the highest possible patient desire for autonomy. In our sample, the scale's internal consistency was satisfying (α = 0.78).

Then, we assessed our primary outcome measure for participation preference: uro‐oncological case vignettes (API‐Uro).^26^ The API‐Uro consists of four vignettes, which describe seven typical decisions that may occur during uro‐oncological treatment. Patients indicate who should make the corresponding decision on a 5‐point Likert scale from (1) “The physician alone” to (5) “The patient alone.” Scores of three indicated a preference for equally shared decisions. A maximum sum score of 35 indicates the highest possible patient desire for autonomy. The internal consistency of the scale was satisfying (α = 0.83). We used the original API case vignettes to assess the validity of the uro‐oncological case vignettes (API‐Uro). The two measures correlated at *r* = 0.568, *p* < 0.01.

For better comparisons between the measures (API generic and API‐Uro), we conducted linear transformations to a scale from 0 to 100. By referring to previous literature,^28^, ^29^ we created a taxonomy for participation preference: Scores <40 indicate a preference for delegating decision making to the physician, and scores ≥40 indicate a preference for participation and autonomy in decision making.

#### 2.2.4 Anxiety

We measured anxiety with the German state version of the State–Trait Anxiety Inventory (STAI),^24^, ^30^ a well‐validated, reliable, and sensitive questionnaire. The scale consists of a 20‐item scale (*α* = 0.93). Patients are asked to report the intensity of their feelings at that moment on a 4‐point Likert scale from (1) “not at all” to (4) “very much so.” Sum scores range from 20 to 80. A cut‐off score of ≥55 is recommended to define clinically significant levels of anxiety in older patient populations.^31^

#### 2.2.5 Personality

To assess the five most robust dimensions of personality (openness, conscientiousness, extraversion, agreeableness, neuroticism), we used the German version of the BFI‐10,^23^ a short version of the well‐established Big Five Inventory (BFI).^18^ The BFI‐10 is a short, sufficiently reliable measure with five two‐item sub‐scales. Responses are coded on a 5‐point Likert scale from (1) “disagree strongly” to (5) “agree strongly.” Sum scores are computed for each scale, with higher values representing a stronger expression of this personality dimension. We conducted linear transformations to a scale from 0 to 100.

### 2.3 Data analyses

All analyses were carried out with Statistical Package for Social Sciences (version 27.0, IBM Corp., 2020). For each measure, we excluded patients with more than 10% missings from the respective analysis. We imputed missing values (≤10%) by subscale mean for participation preference and anxiety. We conducted a drop‐out analysis for all sociodemographic variables to examine potential systematic differences between completers and non‐completers. We compared sociodemographic characteristics for patients who preferred incontinent vs. continent UD with either *χ* ^2^‐tests or independent sample *t*‐tests. Univariate descriptives are reported for anxiety, personality, and participation preferences. To assess differences in decision context (i.e., API and API‐Uro) we conducted splits for patients who preferred to delegate decisions to the physician (delegators) and those who preferred participation in decision making (participators, cut‐off of ≥40) and compared proportions in a contingency table. Then, we conducted group comparisons between patients who preferred continent vs. incontinent UD (independent sample *t*‐tests) for participation preferences, anxiety, and personality traits. We calculated Pearson product–moment correlations between all variables of interest to analyze the association of individual characteristics with uro‐oncological participation preference. All significant variables were then tested as predictor variables in a subsequent multiple linear regression analysis. Statistical significance was set at α = 0.05. Where applicable, we report effect sizes and interpret them based on Cohen's taxonomy.^32^

## 3 RESULTS

### 3.1 Descriptive information

Of the *N* = 191 patients who received a questionnaire, 5.24% were non‐completers. Drop‐out analyses indicated that there were no significant differences between groups. Table 1 summarizes the demographics of the final patient sample (*N* = 180). The sample was primarily German, male, without secondary school education, in a stable relationship, and most had children. The mean age was 68.77 years (SD = 9.28; range 46 to 86). Most patients (65.6%) had a clinical tumor stage cT2 or higher. The majority reported a clear treatment preference (78.9%), and most preferred continent to incontinent forms of UD (44.4% vs. 34.4%), 21.1% did not report a preferred treatment.

**TABLE 1:** Sociodemographic characteristics of the sample split for patients who reported a preference for continent UD or incontinent UD

Characteristics^a^	Total sample (*N* = 180)		Continent UD (*n* = 80)		Incontinent UD (*n* = 62)	
	*M*	SD	*M*	SD	*M*	SD
Age***	68.77	9.28	65.09	8.93	71.11	8.43
Gender	*n*	%	*n*	%	*n*	%
Male	136	75.6	65	81.3	44	71.0
Female	43	23.9	15	18.8	18	29.0
Nationality						
German	171	95.0	75	93.8	61	98.4
Other	9	5.0	5	6.3	1	1.6
Educational level						
University degree	36	20.0	21	26.3	9	14.5
Secondary school education	14	7.8	7	8.8	5	8.1
No secondary school education	120	66.7	50	62.5	42	67.7
No school education	5	2.8	0	0	3	4.8
Employment***						
Retired	124	68.9	40	50.0	52	83.9
Employed	43	23.9	34	42.5	7	11.3
Unemployed	6	3.3	3	3.8	1	1.6
In education	3	1.7	1	1.3	1	1.6
Relationship status						
Living together with a partner	137	76.1	64	80.0	47	75.8
Separated, single or widowed	43	23.9	16	20.0	15	24.2
Offspring						
With children	153	85.0	67	83.8	52	83.9
Without children	26	14.4	12	15.0	10	16.1

^a^ Significant differences in the characteristics between patients who preferred incontinent and continent UD are marked by * and determined either by Pearson's *χ* ^2^‐test or independent sample *t*‐test. Diverging numbers of patients are due to missing values.

^***^ *p* < 0.001.

Many patients reported high levels of anxiety (*M* = 49.57, SD = 11.57), and a third (32.2%) scored above the clinical cut‐off for significant anxiety symptoms. Mean scores for the five personality dimensions were as follows: openness (*M* = 60.52, SD = 24.78), conscientiousness (*M* = 77.11, SD = 18.63), extraversion (*M* = 55.83, SD = 24.14), agreeableness (*M* = 58.10, SD = 18.38), neuroticism (*M* = 47.47, SD = 20.86).

Most patients expressed a strong desire for information (*M* = 91.63, SD = 12.11), but a low desire for autonomy in generic decision making (*M*_API_ = 30.99, SD = 23.71). Participation preference was also low in the case vignettes (*M*_original vignettes_ = 29.82, SD = 13.56; *M*_API‐Uro_ = 28.51, SD = 14.05). The two measures correlated at *r* = 0.568, *p* < 0.01; the means did not differ significantly (*p* = 0.229).

Most patients (62.2%) preferred to delegate decision making to healthcare professionals for generic decision making (API), and even more (75.6%) for uro‐oncological decisions (API‐Uro). A split for delegators vs. those who preferred participation (cut‐off of scores ≥40) classified most patients as delegators for both generic and uro‐oncological decision making (58.1%). Still, over a quarter of patients (28.8%) preferred participation on one, but not the other measure (for more details see Table 2).

**TABLE 2:** Contingency table for patient‐reported participation preferences for generic decision making (API) and on the uro‐oncological case vignettes (API‐Uro)

Uro‐oncological Participation Preference (API‐Uro)	Generic participation preference (API)		
	*N* (%)	*N* (%)	*N* (%)
	Delegators	Participators	Total
Delegators	93 (58.1)	35 (21.9)	128 (80.0)
Participators	11 (6.9)	21(13.1)	32 (20.0)
Total	104 (65.0)	56 (35.0)	160 (100)

*Note*: Cut‐off for participators ≥40 on the respective measure.

### 3.2 Interindividual differences in treatment preferences

Patients who preferred continent to incontinent UD showed no differences in their generic participation preference (API, *p* = 0.48), their scores on the uro‐oncological case vignettes (API‐Uro, *p* = 0.94), or their anxiety (*p* = 0.09). Table 3 summarizes the group comparisons for participation preferences, anxiety, and personality. In their five‐factor personality profiles patients who preferred continent UD scored higher in conscientiousness (*M* = 81.33, SD = 15.75) than patients who preferred incontinent UD (*M* = 72.18, SD = 21.16, *t*[139] = −2.94, *p* < 0.01). There were no significant differences for the other dimensions of personality. Patient groups did not differ on anxiety or participation preference.

**TABLE 3:** Group comparisons for patients who preferred continent versus incontinent UD: means, standard deviations, *t*‐tests, and effect sizes

Measures	Continent UD (*n* = 80)		Incontinent UD (*n* = 62)		*t(df)*	*p*	Cohen's *d*
	*M*	SD	*M*	SD			
Participation preference							
Generic participation preference (API)	32.15	23.00	29.24	24.19	−0.715(133)	0.476	
Uro‐oncological participation preference (API‐Uro)	26.98	13.48	26.81	14.30	−0.071(132)	0.943	
Anxiety (STAI)							
STAI state	47.64	11.47	51.10	11.93	1.69(131)	0.093	
Personality (BFI‐10)							
Openness	64.87	23.09	57.99	27.58	−1.61(138)	0.111	
Conscientiousness	**81.33**	**15.75**	**72.18**	**21.16**	**−2.94(139)**	**0.004**	**−0.50**
Extraversion	59.49	22.31	55.85	25.28	−0.91(139)	0.365	
Agreeableness	58.23	18.87	59.07	16.19	0.28(139)	0.779	
Neuroticism	44.46	20.09	47.18	19.79	0.80(139)	0.424	

*Note*: Significant group differences are marked in bold print.

### 3.3 Correlates and predictors of uro‐oncological participation preference

We conducted correlation analyses for all variables to explore the relationships between participation preferences, anxiety, and personality. Exact statistics are reported in Table 4. We observed no significant correlations between generic participation preference (API) and anxiety or personality (*p*s > 0.05) except for agreeableness (*r* = −0.166, *p* < 0.05). Our results indicate small effects for uro‐oncological participation preference (API‐Uro) and anxiety and neuroticism: Higher uro‐oncological participation preference (API‐Uro) correlated with increased anxiety (*r* = 0.202, *p* < 0.05) and higher neuroticism (*r* = 0.180, *p* < 0.05).

**TABLE 4:** Correlation coefficients for participation preference, anxiety, and five‐factor personality

Variable	1	2	3	4	5	6	7	8
1. Generic participation pref.	‐							
2. Uro‐oncological participation pref.	0.447**	‐						
3. Anxiety	0.142	0.202*	‐					
4. Openness	0.045	−0.016	−0.078	‐				
5. Conscientiousness	0.068	−0.098	0.025	0.240**	‐			
6. Extraversion	0.015	−0.120	−0.299**	0.146	0.114	‐		
7. Agreeableness	−0.166*	−0.032	−0.040	0.160*	0.104	−0.158*	‐	
8. Neuroticism	0.097	0.180*	0.369**	−0.040	0.062	−0.209**	−0.084	‐

*Note*. Correlations reported as Pearson's *r*. *p < 0.05, **p < 0.01, two‐tailed. Effects that are small or larger are marked in bold print. Information Seeking, Generic Participation Pref. (Autonomy Preference Index); Uro‐Oncological Participation Pref. (API‐Uro); Anxiety (STAI State); Openness, Conscientiousness, Extraversion, Agreeableness, Neuroticism (BFI‐10).

We conducted multiple linear regression analyses for uro‐oncological participation preference (API‐Uro) and included anxiety and neuroticism as predictors. The regression model explained 3.7% of variance, model fit *F*(1,156) = 6.936, *p* < 0.01, with an *R* ^2^ of 0.043 (∆*R* ^2^ = 0.037). Only anxiety was a significant predictor of uro‐oncological participation preference (*β* = 0.207, *t*[156] = 2.634, *p* < 0.01).

## 4 DISCUSSION

Although health care providers generally agree on the importance of SDM, little is known about psychological patient characteristics that should be considered in this process. Our findings provide insight into the role of psychological patient characteristics (i.e., anxiety and five‐factor personality traits) for SDM on two dimensions: patient‐reported preferences for treatments and participation preferences. First, our results on patient‐reported treatment preference emphasize the relevance of personality. As expected, individuals who reported lower contentiousness were more likely to prefer less complex treatment methods associated with a less intensive rehabilitation process (incontinent UD). Our hypothesis that openness might be relevant was not confirmed. While we found no significant effect, our descriptive data (as shown in Table 3) indicate that individuals with higher openness tend to prefer more complex treatment methods (continent UD), which may be more suitable to safeguard their active lifestyle. Second, our results on patient‐reported participation preference provide evidence that anxiety may drive the motivation to be more involved in pertinent decisions. Decision context is clearly relevant for participation preference. Even though mean values for generic and uro‐oncological decision making were similar, we found substantial differences in proportions between participators and delegators for each measure as well as differences regarding associated factors.

Our results strengthen and expand previous findings on the effects of personality on SDM.^17^ We identified conscientiousness as an important personality dimension regarding patient‐reported treatment preference. This is well in line with previous findings that established a significant link between conscientiousness and patient adherence to treatment.^33^ Individuals with higher agreeableness reported lower generic participation preference, with a small effect size. This fits with literature that suggests patients' fear of being labeled a ‘bad patient’ can limit active engagement and predict lower participation preference.^34^ It is possible that we did not find the same effect for uro‐oncological participation preference because these decisions are more pertinent to the patients and their uro‐oncological healthcare choices have consequences for patients' health. We encourage future studies to replicate and extend these findings on personality.

Patients in our sample reported a high rate of clinical anxiety, which replicated previous findings with uro‐oncological patients.^7^ High levels of anxiety may drive the motivation to be engaged in SDM for specific decisions relevant to the patient. In our regression model for uro‐oncological participation preference, anxiety explains only a small proportion of variance. While this leaves a large proportion of variance unaccounted for, participation preference is a multifaceted phenomenon, and one cannot expect a single factor to explain large proportions of variance. Moreover, our results replicate recent findings on anxiety and participation preference for COVID‐19 related decision making.^13^

Our results support and extend our previous finding that individuals who experience symptoms of anxiety and depression before a medical consultation are more concerned and conflicted about their treatment decisions in an independent sample.^7^ More anxious individuals may already feel conflicted about treatment options before the consultation and consequently experience a more vital need to address these concerns and worries, resulting in a higher participation preference. However, higher participation preference does not necessarily entail more participation in decision making; a mismatch between the two is not uncommon.^35^ To achieve concordance between preferred and perceived level of involvement and thus higher patient satisfaction, physicians should facilitate a discussion about participation in SDM with their patients. While a recent study on patient participation in urology suggests high levels of overall patient involvement and satisfaction, many patients still report not having been explicitly asked about their preferred involvement in decision making.^36^ Patients' decision making preferences are a valuable information and should be regularly assessed and applied in clinical practice.^9^ Our results demonstrate that physicians should especially support and engage more anxious patients during decision making. It is well known that effective responses to patients' informational and emotional needs can reduce distress in cancer patients.^14^

### 4.1 Study limitations

Our conclusions need to be considered in light of some limitations. First, our sample size is limited, even though it is in line with previous studies on muscle‐invasive bladder cancer^20^ and sufficient to detect medium effect sizes with an independent sample *t*‐test. However, we may have been unable to identify smaller effects for sub‐group comparisons (e.g., an association between openness and patient‐reported treatment preferences). Also, while the sample characteristics (primarily older males) are typical for bladder cancer patients, the limited proportion of younger and female patients decreases the generalizability of our findings to other patient groups. Furthermore, as male cancer patients are less likely to report anxiety than females,^37^ we may have underestimated anxiety in our sample. Still, the anxiety rate is in line with comparable studies on cancer patients that include more females.^38^ Also, the multicenter approach contributes to the generalizability of our findings. While other aspects of our study samples' composition (e.g., demographics such as nationality, relationship status, and having children) could be relevant to patients' perception of treatment alternatives and their preferences regarding choice of UD, we found no significant differences for patients who preferred continent versus incontinent UD for any of these characteristics. However, other characteristics that may have influenced patient preferences, like patients' pre‐consultation level of information on treatment options, was not controlled for. Future studies may opt to consider this.

Second, our results are based on self‐report. This is the most common method, and self‐report tests and case vignettes are widely used to determine patient participation preferences. But the use of hypothetical case vignettes has been evaluated critically as there can be a gap between hypothetical and actual preferences.^28^ However, the uro‐oncological case vignettes (API‐Uro) we used to describe typical decisions for the oncological condition that our patient sample was affected by, thus minimalizing a potential empathy gap for the scenario. Similar procedures have contributed valuable information to the literature.^13^

### 4.2 Conclusion and clinical implications

Treatment decisions in oncology are often highly preference‐sensitive, as they are very invasive and involve a significant amount of uncertainty. The choice for the best fit of UD, faced by patients with muscle‐invasive bladder cancer, is a prime example of this. Our results demonstrate that different patients have different preferences, and personality traits and emotions such as anxiety are important factors for both treatment and participation preferences.

Screening patients for relevant aspects of personality and emotional distress may help healthcare professionals identify those who are more hesitant about complex treatment options and those who want more participation in their medical decisions. This would benefit the discussion on treatment approaches and foster patient‐centered care. An effective way to increase patient support and facilitate patient engagement in decision making can be to use decision aids^39^; available decision aids for urological cancer entities have recently been summarized and evaluated.^40^ At the same time, some patients may benefit from psychosocial interventions to reduce negative affect and increase support in decision making.^41^

Our results highlight the need for a systematic assessment of patient‐reported preferences of specific treatments and also of their preferences concerning participation in the clinical setting. This may foster patient engagement. Most importantly, anxiety needs to be assessed and addressed in the medical consultation to enable the professional to relieve the emotional distress patients experience during cancer treatment.

## CONFLICT OF INTEREST

The authors have no conflict of interest to declare.

## AUTHOR CONTRIBUTIONS

A.K. Köther: study conception and design, data management, analysis, and interpretation of data, manuscript writing, manuscript editing. B. Büdenbender: study conception and design, manuscript editing. B. Grüne: acquisition of data, manuscript editing. S. Holbach, J. Huber, N. von Landenberg, J. Lenk, T. Martini: acquisition of data. M.S. Michel: acquisition of funding, supervision. M.C. Kriegmair: acquisition of funding, study conception and design, acquisition of data, supervision, manuscript editing. G.W. Alpers: acquisition of funding, study conception and design, interpretation of data, supervision, manuscript editing and revision.

## ETHICS STATEMENT

Ethical approval for the study was received from the research ethics committee of the Medical Faculty of Mannheim of the University of Heidelberg (MA‐2019‐727 N). Patients provided written informed consent.

## Data Availability

The data used for this study are available upon request and have been deposited on MADATA (University of Mannheim) Research Data Repository at 10.7801/389 ^42^ and will be made available by the authors, without undue reservation, to any qualified researcher.
